# Using experience-based co-design approach to address mental health issues among healthcare workers and leaders in long-term care

**DOI:** 10.3389/fmed.2025.1535017

**Published:** 2025-04-04

**Authors:** Sheila A. Boamah, Mawukoenya Theresa Sedzro, Humayun Kabir, Brenda Vrkljan, Scott Mitchell, Laura De La Torre Pérez, Marilina Santero

**Affiliations:** ^1^School of Nursing, Faculty of Health Sciences, McMaster University, Hamilton, ON, Canada; ^2^Faculty of Health Sciences, Western University, London, ON, Canada; ^3^Department of Health Research Methods, Evidence, and Impact, Faculty of Health Sciences, McMaster University, Hamilton, ON, Canada; ^4^School of Rehabilitation Science, Faculty of Health Sciences, McMaster University, Hamilton, ON, Canada; ^5^Centres for Learning, Research and Innovation in Long-Term Care at the Schlegel-UW Research Institute for Aging, Waterloo, ON, Canada; ^6^Methodology of Biomedical Research and Public Health PhD Program, Universitat Autónoma de Barcelona, Barcelona, Spain; ^7^Centro Cochrane Iberoamericano, Sant Antoni Maria Claret, Barcelona, Spain; ^8^Department of Paediatrics, Obstetrics and Gynaecology, Preventive Medicine and Public Health, Universitat Autònoma de Barcelona, Barcelona, Spain

**Keywords:** burnout, mental health, co-design, healthcare workers, long-term care, retention, experiential research

## Abstract

**Introduction:**

Burnout remains a critical issue within the long-term care (LTC) sector, underscoring the urgent need for early detection and prevention strategies targeting the healthcare workforce. This paper aimed to describe the processes of uncovering the underlying drivers of burnout and distress among LTC workers, providing a foundational understanding to inform the development of a Burnout Assessment Tool (BAT).

**Materials and methods:**

Using an experience-based co-design approach, 11 focus groups were held with a purposive sample of 24 Ontario LTC workers between July 2023 and October 2024. Each session, lasting 2 h, included 4–5 participants representing a diverse range of professional and occupational roles, including personal support workers, nurses, social workers, and administrators or leaders. The objectives of this approach were two-fold: (1) to explore the work-related experiences of LTC workers; and (2) to examine their perceptions of existing burnout and distress tools, including the Maslach Burnout Inventory-Human Services Survey, to assess their relevance and applicability within the LTC context. Each focus group session was audio-recorded and transcribed verbatim. An iterative process generated codes from the transcripts that culminated in a thematic framework of key findings.

**Results:**

Four interrelated themes emerged: (1) challenges inherent in the LTC work environment; (2) the impacts of workplace pressures on employee health and well-being; (3) managing psychosocial risk factors in the workplace; and (4) the need for a context-specific tool to assess burnout in LTC settings. Participants provided in-depth perspectives on their roles within the LTC sector, highlighting the profound impact of burnout on their well-being and the persistent challenges they face in their work environment.

**Discussion/conclusion:**

The findings highlight the pressing need for tailored systemic interventions to effectively address burnout and moral distress among LTC workers and leaders. By employing a co-design approach, this study offers critical insights into the lived experiences of these workers, informing the development and refinement of burnout assessment tools to better reflect the unique needs of this workforce. Developing a BAT, co-created by and for LTC workers, coupled with robust support systems, is crucial to addressing the rising distress and fostering resilience within this vital sector.

## 1 Introduction

Burnout is defined as a condition of fatigue characterized by cynicism toward the worth of one’s profession and ability to execute tasks (1). The 11th revision of the International Classification of Diseases (ICD-11) recognizes burnout as a syndrome resulting from prolonged and inadequately managed workplace stress (2). The inclusion of burnout in this latest edition signals the rising importance and implications on workers, where systemic changes in workplace practices, policies, and culture are critically needed (3, 4).

Burnout is a pressing concern in the health care sector, especially among healthcare workers (HCWs) in long-term care (LTC). Heightened levels of emotional exhaustion, depersonalization, and reduced personal accomplishment have been reported amongst workers in this sector; some exceeding 40% of the workforce (5, 6). Burnout not only reduces worker productivity, job satisfaction, and retention (7–10), but also has profound implications for psychological health (e.g., insomnia, depression) and physical well-being (e.g., gastrointestinal issues, hypertension, chronic fatigue) (11). While HCWs across all sectors experienced significant stress during the COVID-19 pandemic, those in LTC faced additional hardships, including job insecurity, persistently low wages, and precarious employment conditions (12). Unlike their counterparts in hospital settings, LTC workers often develop close, personal relationships with residents, intensifying moral distress and emotional exhaustion (12). The repercussions of the pandemic were particularly detrimental to the morale of LTC workers (12, 13), compounding sector-specific challenges and further exacerbating burnout.

Prior to the pandemic, multiple work-specific stressors had already been linked to burnout in LTC, including, but not limited to, heavy workloads, time pressure, role conflict and ambiguity, as well as physical exhaustion (14, 15). Yeatts (16) identify factors such as workplace design (e.g., workload, empowerment, role conflicts), interpersonal relationships (e.g., management and co-worker support), and individual characteristics (e.g., age, and length of employment) as key contributors to burnout among HCWs in LTC. While examining organizational/workplace, interpersonal, and personal factors captures many relevant variables, additional factors may be crucial to fully understanding burnout in LTC settings.

The increasingly complex medical needs of LTC residents have placed additional pressure on HCWs augmenting their vulnerability to burnout. This complexity has intensified the emotional strain on HCWs. Although the medical needs and associated burden of caring for LTC residents is increasing, staffing levels do not match the demand for this care (17). As such, addressing burnout among HCWs in this setting is both urgent and critical (17–19). In response, the current study – conducted as part of the *Healing the Healers’ (HH) project* – adopted a co-design approach to explore the experiences of LTC workers focusing on meso- and macro-level factors contributing to burnout and mental distress. Using this approach, the study aims to inform the development of a burnout assessment tool (BAT) tailored to LTC. By highlighting key challenges and opportunities encountered across these roles, these insights will guide both practice and policy to address this need with the ultimate goal of ‘healing the healers’ in LTC.

Co-design approaches are increasingly common in health innovation and reform, empowering stakeholders to collaboratively shape a shared agenda that fosters collective action and practical solutions (20, 21). Central to co-design is the emphasis on collaborating with, rather than designing for, end users. At its core, co-design redefines the relationship between researchers and participants by flattening hierarchies to harness insights from lived experiences. A foundational principle of co-design is that participants are viewed as the experts, rather than the researcher (22). In healthcare, co-design can inform and transform the relevance of strategic outcomes. Several healthcare reformers, including Sanders and Stappers (22) and Batalden et al. (23), have highlighted co-design as a method of harnessing the collective intelligence and knowledge of diverse stakeholders. Evidence has shown the effectiveness of this approach in developing healthcare products and systems (24, 25).

The current study employed a co-design approach for three main reasons. First, its participatory approach positions LTC workers as research experts thereby ensuring their involvement from the project’s inception to its conclusion. Second, while burnout and mental health challenges are widespread issues within the healthcare sector, co-design enabled the research team to seek a nuanced understanding of distress and burnout from the perspective of LTC-HCWs. Lastly, this approach facilitates actionable research, bridging the gap between theory and practice (26).

**Figure 1:**
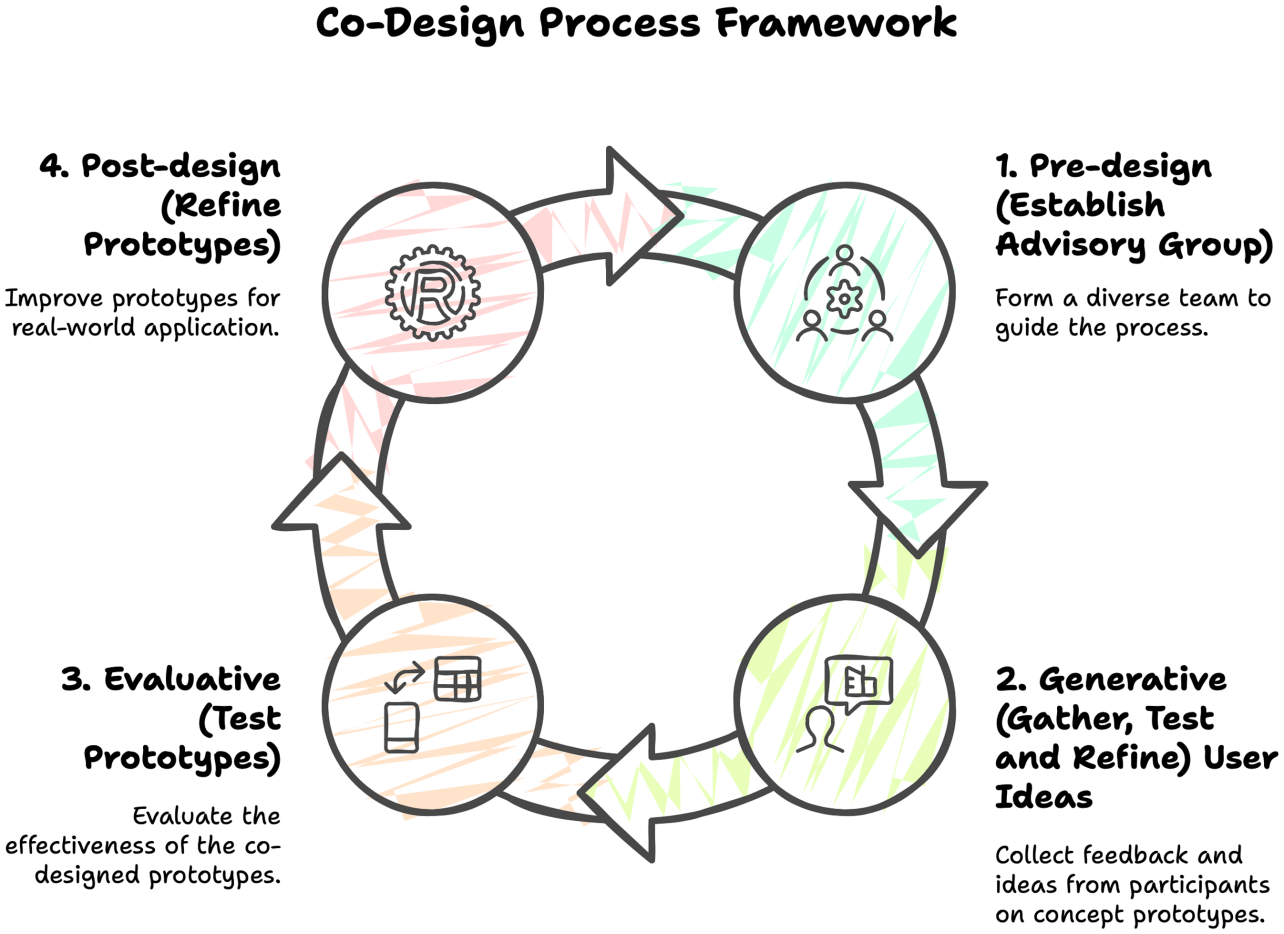
The four-phase co-design process.

To understand the experiences of those working in this sector, we adopted Sanders and Stappers’ (27) “co-design process framework.” This framework involves users and researchers across the *pre-design, generative, evaluative*, and *post-design* phases, which involve communal learning, collective knowledge co-design, and multidisciplinary collaboration across a range of HCWs from diverse backgrounds (Figure 1).

## 2 Materials and methods

### 2.1 Description of the process

Following Sanders and Stappers’ (27) four-phase process, we fostered collective creativity through structured co-design activities involving users, researchers, and other stakeholders as design partners, informants, or testers. The first phase of the co-design process framework (pre-design) involves establishing a multidisciplinary advisory group to incorporate a diversity of experience and expertise throughout the process. In phase two (generative), ideas are gathered from users based on concept prototypes developed by researchers. Phase three (evaluative) involves testing the co-designed prototypes. The final phase (post-design) focuses on refining a prototype ready for further testing in real-world settings (see Table 1 for a description of the phases).

**Table 1:** Description of the co-design methodological framework.

Phases	Purpose	Activity	Description
1. Pre-design	To understand people’s experiences in the context of their lives - past, present and future	Recruitment of expert panel members	To prepare people to participate in co-designing. Experienced workers within the LTC sector are sought to become key stakeholders in charting a new path to addressing mental health issues among HCWs.
2. Generative	To produce ideas, insights and concepts that may then be designed and developed	Focus group meetings	Deciding on what will be helpful, usable, and desirable. Meetings held with various participants to discuss: (1) issues within LTC, and (2) best approaches to adapting some existing questionnaires to produce a single tool. The final product is shaped by the experiences of the expert panel.
3. Evaluative	To assess, formatively or summatively, the effect or the effectiveness of products, spaces, systems or services	Province-wide roll out of assessment tool	Assessing the tool’s usefulness, usability, and desirability; Identification of problems; Measurement of effectiveness Administer tool to the larger population of HCWs in LTC to evaluate the prevalence of mental ill-health.
4. Post-design	To understand people’s experiences in the context of their lives - past, present and future	Pilot intervention program	The results of both generative and evaluative phases will be consolidated to create an ideal approach to managing mental health issues among HCW in LTC. This will be developed into an intervention program that will be piloted in a LTC home.

In this paper, the co-design process undertaken for the HH project is described. Preliminary findings from the first two phases of this process are described with follow up phases disseminated in subsequent articles.

#### 2.1.1 Phase 1 (pre-design): establishing an expert panel

In the pre-design phase, participants with relevant expertise were carefully selected through targeted outreaches, collaborations with our LTC partners, and inclusive recruitment strategies. Roles and responsibilities were clearly defined and assigned across project stages to facilitate efficient and effective decision-making (28, 29). This approach allowed for selective involvement, meaning that not all advisory group members needed to participate in every activity or phase of the process at the same time. Eligibility criteria included various LTC workers in Ontario, such as personal support workers (PSWs), nurses, social workers, and LTC leaders (administrators and managers). To promote a diverse participant pool and facilitate meaningful discussions, individuals were recruited across differing roles, years of experience, and geographical regions. Screening criteria also required familiarity with the 13 factors outlined in the National Standard of Canada for Psychological Health and Safety in the Workplace (“the Standard”)—a set of voluntary framework providing guidelines, tools, and resources to support mental health and prevent psychological harm at work (30). These factors include organizational culture, psychological and social support, clear leadership and expectations, recognition and reward, workload management, and physical safety protection. Recruitment efforts targeted both rural and urban communities across Ontario. Emails, workshops, and webinars were used to reach LTC homes and organizations, while flyers were distributed via a snowball sampling approach to broaden the participant pool. Interested individuals were screened to confirm their work experience in Ontario care facilities and their roles as HCWs or leaders. Social media platforms, such as Twitter, were also leveraged for dissemination. Recruitment materials were written in plain English, using gender-neutral language and sensitivity to ethnicity, race, and key intersectional factors.

#### 2.1.2 Phase 2 (generative): gathering, testing and refining insights, ideas, and concepts

In accordance with the *generative* phase of co-design, we sought out to understand HCWs’ experiences of the work environment and the LTC system. This phase began with a series of focus group discussions, facilitated by the lead researcher, aimed at exploring various concepts, including the challenges and opportunities in LTC and potential solutions for preventing burnout and mental distress. Between July 2023 and October 2024, a total of 11 focus group sessions were conducted, both in-person and via Zoom, to accommodate geographic and scheduling constraints of the 24 participants. The initial six sessions, each lasting 2 h with 4–5 participants, were followed by five corresponding follow-up sessions with the same groups to build on earlier discussions. To address potential power dynamics, we conducted two sub-group sessions focused on specific HCWs, such as PSWs, enabling us to gain a deeper understanding of their unique needs and challenges.

The first round of focus groups, “Framing the Problem,” explored LTC workers’ experiences, focusing on challenges in the work environment and factors contributing to HCW burnout. The expert panel (participants) shared insights on working in LTC, their experiences, including working during COVID-19, and perspectives on the co-design process. The second round, “Exploring Solutions and Approaches,” delved deeper into these challenges by identifying various macro, meso, and micro-level factors contributing to burnout and explored solutions. Participants explored ideas and a shared vision for innovative approaches to address the 13 psychological factors outlined in “the Standard” for workplace health, including workload management, engagement, balance, psychological demands, civility and respect, and growth and development (30). These discussions also sought to inform mental health assessment tools, focusing on anxiety, depression, and burnout among workers in LTC.

**Figure 2:**
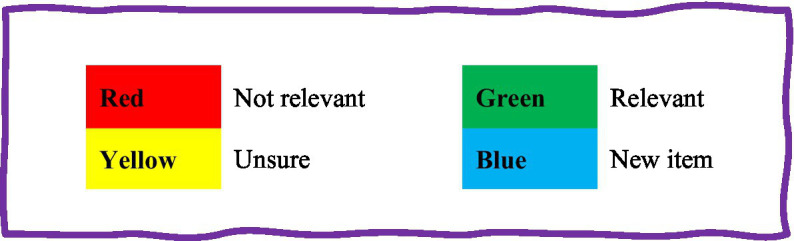
Guide for colour coding questions in the adapted questionnaire, Healing the Healers (HH) project.

As part of the *generative* phase of the co-design, the most effective elements of group discussions are consolidated and refined through iterative cycles into a single idea or tool (prototype). In this study, the tool development and adaptation process commenced with the selection of rigorously validated psychological instruments designed to assess mental health in the general population. Examples of questionnaires used included the Patient Health Questionnaire (PHQ-4), General Anxiety Disorder (GAD-7) scale, Moral Injury Symptom Scale-Health care Professionals, Beck Depression Inventory, Maslach Burnout Inventory, and Brief Resilience Scale (BRS-6). The PHQ-4, for instance, is a 4-item Likert scale assessing psychological distress, including anxiety and depression, while the BRS-6 is a 6-item scale measuring resilience in the face of stressful events. To ensure the relevance of these questionnaires for the LTC context, participants were invited to critically evaluate each instrument, assessing readability, usability, and applicability within their work environment. This review process was conducted individually and then collaboratively during a second-round focus group, with a particular focus on both the substantive relevance and effectiveness of questionnaire items in capturing the intended constructs (e.g., burnout and mental distress) within the LTC setting, as well as the clarity and format of each instrument. Using a color-coded ranking system (Figure 2), participants discussed the importance of each item, making adjustments as necessary to enhance clarity and relevance. This assessment aimed to inform co-design discussions and explore interventions for reducing burnout and improving retention. Key points from the discussions were consolidated at the end of each focus group.

### 2.2 Analysis of data

Focus group discussions were recorded for reference, imported and transcribed using Otter.ai software, and thematically analyzed through the Delve tool. The Otter.ai software facilitated the correction of transcription errors by synchronizing text with speech, ensuring accuracy. Once cleaned, the transcripts were uploaded into Delve, an online platform designed for the efficient analysis of large qualitative datasets. Using Delve, we systematically coded the data to identify key themes. Our analytic approach adhered to Braun and Clarke’s (31) six-phase model, which encompasses data familiarization, initial coding, theme identification, review, definition, and report writing. Using directed qualitative content analysis technique, two researchers independently and collaboratively reviewed audiotaped recordings, personal memos, and reflections from the co-design process. The qualitative analysis in Delve involved the following steps. First, the data organization process employed tags and categories, with individual data points labeled with tags and related points grouped into categories. Second, the coding process began with the development of an initial coding framework crafted through careful reading of interview transcripts. Third, the expert panel was guided by the research lead to generate codes that reflected key issues with concise words or phrases from the transcripts. An engagement-driven coding approach was used, whereby the entire transcript of each participant was analyzed to capture key aspects of their lived experiences in depth. This iterative process involved going back and forth between transcripts and initial codes and developing higher level categories of such codes. The next step involved identifying patterns by sorting codes into initial themes that emerged inductively based on their significance in the transcripts, following a criterion of keyness.

To ensure rigour, the research team engaged in member checking by ensuring there was consensus on major themes. Relationships among codes, themes, subthemes, and data were analyzed. Two researchers independently reviewed the identified themes and subthemes, consulting with the broader research team and participants by sharing a summary for feedback. Participant feedback was then incorporated to refine the final themes and subthemes, ensuring coherence and accuracy. Additionally, participants were offered an extended opportunity to provide further verbal or written feedback within a two-week period. This collaborative process culminated in a well-defined thematic framework, offering a thorough and dependable representation of the qualitative data analysis conducted using Delve.

## 3 Results

**Figure 3:**
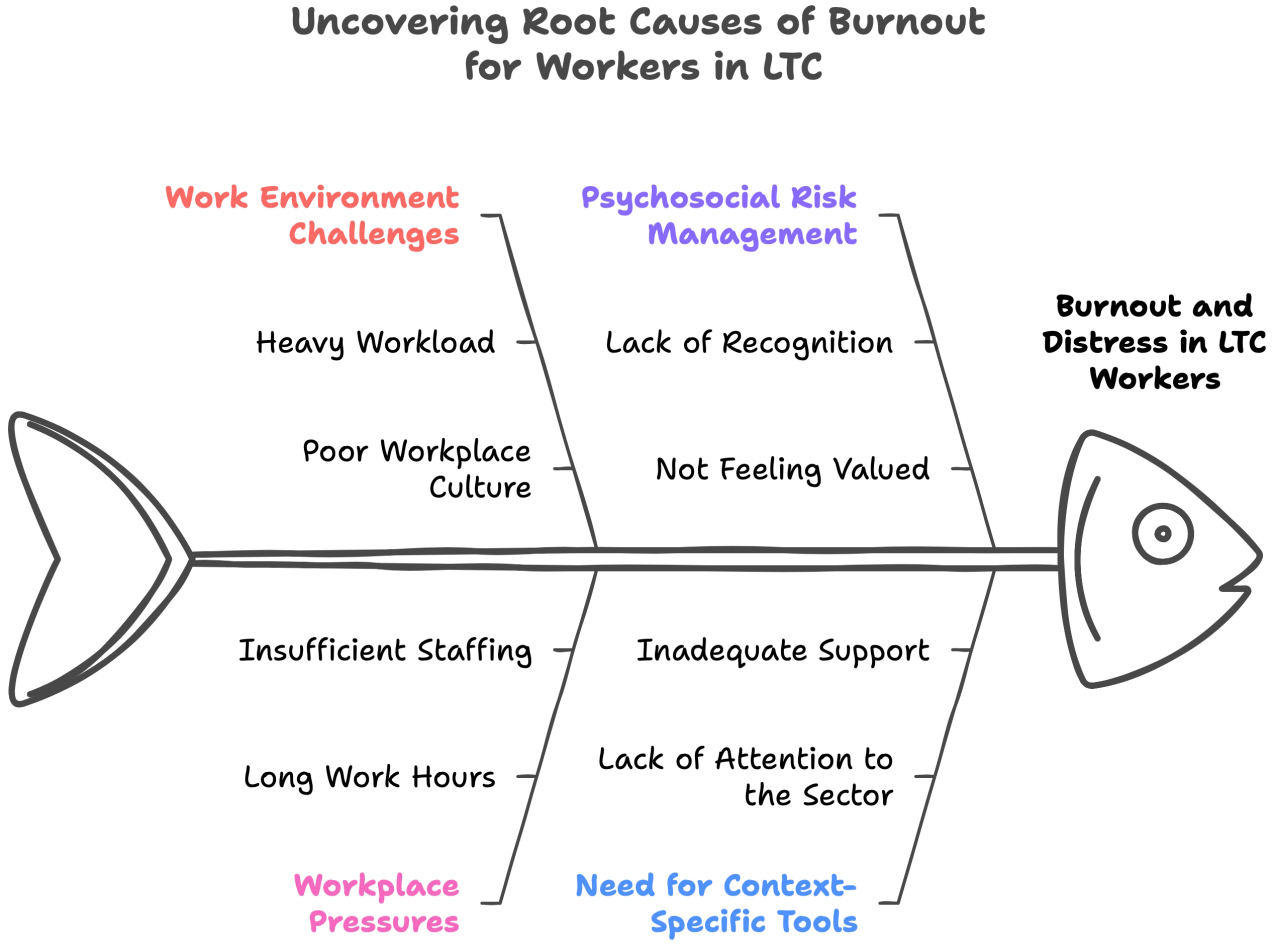
Contributory factors associated with burnout.

Overall, 24 HCWs and leaders participated in the co-design process (see Table 2 for participant demographic characteristics). To gain insight into their experiences in LTC, the preliminary findings offer an overview of emerging themes, challenges, and potential interventions. These insights are categorized into four key themes: (1) *Challenges inherent in the LTC work environment*; (2) *The impacts of workplace pressures on employee health and well-being*; (3) *Managing psychosocial risk factors within the workplace*; and (4) *The need for a context-specific tool to assess burnout in LTC settings* (Figure 3).

**Table 2:** Participant demographic characteristics.

Characteristic	Sample size
Sex	Male: 3
	Female: 21
Age range	27–71 years
Race	White: 16
	Other: 8
Roles	Leader (Manager/Administrator): 5
	Nurse: 5
	Personal Support Worker (PSW): 10
	Social Worker (SW): 2
	Horticultural Therapist (HT): 1
	Behavioural Therapist (BT): 1
Years of experience in active role	1–30 years

### 3.1 Challenges inherent in the LTC work environment

Participants across diverse roles in the LTC sector highlighted shared challenges rooted in both internal (meso-level) and external (macro-level) factors. Meso-level issues included staffing shortages, management practices, organizational culture, and relationships with residents/clients, and their families. Macro-level frustrations stemmed from dissatisfaction with regulatory controls, healthcare policies, economic pressures, and societal attitudes toward care providers. A recurring theme in all focus groups was the pervasive stress experienced by HCWs at all levels, including leaders (managers and administrators). Key contributors included resource limitations (e.g., lack of personal protective equipment [PPE]), unsafe/unpredictable work environments, heavy workloads, and the lingering impact of COVID-19. External pressures, such as negative media portrayals of LTC and inconsistent directives from regulatory bodies, were particularly identified as amplifying internal challenges. These interwoven factors collectively diminished morale and compounded the strain on the LTC workforce.

Participants expressed how the unpredictability of the LTC work environment significantly heightened stress among HCWs and created a chaotic atmosphere, often leaving staff frustrated by the need to justify decisions that conflicted with frontline realities. The COVID-19 pandemic magnified these pressures, introducing new safety protocols and compounding stress and exhaustion among HCWs. One participant (Social Worker, Female) reflected: *“When COVID happened, that was amplified by a million...I was on call 24/7, 365 [days a year].”* Another participant (Manager, Male) recounted similar challenges: *“While some homes may have gone through single outbreaks, me and my colleagues at the time, we went through all outbreaks. So in the first year, I think I remember looking at it and I spent 200 days out of 365 days in COVID outbreaks, going from home to home trying to help support through different waves.”* The combination of systemic and pandemic-related challenges underscored the relentless demands placed on LTC workers.

HCWs expressed pervasive exhaustion, while leaders described their struggles to sustain staff morale amidst a fragmented sense of community. Both leaders and frontline workers reported feeling a lack of support, reflecting the widespread challenges faced across roles, as one participant (Nurse, Female) explained: *“The staff...are burnt out and looking to management for support, but management themselves are burnt out.”* Another participant (Manager, Female) added: *“I know PSWs more so than nurses are facing violence every day because they are doing the day-to-day care. It’s difficult with dementia.”* Caring for residents with complex needs compounded these challenges. Many HCWs described juggling diverse responsibilities, from personal care to assisting with recreation and mealtime. This versatility often required creativity within resource constraints. A participant (Social Worker, Female) captured this sentiment: *“There are never enough hours in the day...you have got to be a little bit of a nurse, a little bit of a kitchen person.”* This reflects the broader challenge of balancing competing demands where time and formalized roles fall short. Despite their unwavering commitment to resident care, participants frequently felt undervalued and mistreated, with limited recognition for their efforts. Experiences of job loss and neglect from management deepened frustration and disillusionment. As one participant (Manager, Female) lamented: *“All those years of working so hard...I devoted my life to you guys.”* The pandemic further introduced profound emotional and logistical challenges.

### 3.2 The impacts of workplace pressures on employee health and well-being

Persistent staffing shortages, fear of contracting the virus, strict safety protocols, and isolation—both for residents, restricted from visitors, and for workers, distanced from their families—added to the strain. Participants expressed deep concern about the toll of stressful working conditions on their health and well-being, noting issues such as absenteeism, turnover intentions, and the emotional labor inherent in their roles. These factors perpetuated a cycle of fatigue, with burnout emerging as a central theme. HCWs described its detrimental effects on their physical health, including illness, exhaustion, and diminished work capacity. Burnout was often likened to a sense of drowning. One participant (Manager, Female) explained: *“It’s very much like you are drowning. And I know the frontline felt like that, too.”* Another participant (Horticultural Therapist, Female) highlighted the toll of contracting COVID-19: *“The worst viruses I’ve ever had in my life.”* This emotional strain was intensified by a sense of blame, particularly when managing health and safety responsibilities in an already overstretched healthcare system.

Guilt and moral injury were recurring themes, as HCWs struggled with taking sick leave or considering leaving their roles due to burnout. Participants frequently reported experiencing moral distress, particularly due to the disproportionate number of resident deaths they witnessed. One person (PSW, Female) stated: *“You might not say the word mental health, but it does affect us. We might not call it, you know, cause the word is used very loosely, but that affects you because of the sense of guilt.”* Another (Manager, Male) reflected: *“But you know, the toll it’s taken on individuals, whether you can or not, is pretty deep. There’s a lot of PTSD [post-traumatic stress disorder] among a lot of different team members that I’ve seen and experienced, and even personal experience, where you feel for everything you have gone through, and if you are not in long term care, you do not fully know.”* The emotional burden of caring for residents, meeting family expectations, and navigating bureaucratic challenges further compounded feelings of worthlessness and overwhelm. Additional factors such as poor organizational leadership, time constraints, an inability to provide quality care, and mistrust further eroded their psychological health and well-being.

Staff turnover emerged as a critical issue, exacerbated during the pandemic. Burnout drove some HCWs to leave the field entirely, creating recruitment and retention challenges. One participant (PSW, Female) recounted: *PSWs are leaving because of all this stress and are quitting, going to do a different job.’* New personnel often struggled to adapt to the demanding LTC environment, further straining already limited resources. The combined pressures of inadequate staffing, resource constraints, and regulatory demands intensified burnout for both frontline workers and leaders. Participants emphasized the urgent need for systemic interventions to support HCWs, mitigate burnout, and foster sustainability within the healthcare sector.

### 3.3 Managing psychosocial risk factors within the workplace

The exploration of psychosocial risk factors contributing to burnout among HCWs in the HH project revealed a critical theme: the lack of organizational recognition and implementation of supportive measures. Most participants, regardless of their work setting, reported an absence of acknowledgment and action from employers. As one participant (Social Worker, Female) remarked, *“I cannot say that I worked for organizations that acknowledged these [the 13 psychological factors for workplace health] at all.”* Expanding on this, they added, *“Where I was prior to here [current organization], they talked the talk but did not walk the walk. They would say all these things were important, but they were not.”* Organizational change was identified as a vital step in addressing these risks. Participants highlighted efforts to rebuild organizational structures through staff engagement and collaborative decision-making. However, significant challenges persisted, with many attributing these difficulties to external factors such as legislative constraints, public health guidelines, and resource limitations. A recurring disconnect between organizational rhetoric and practice was evident, as employers often professed to prioritize factors like workload management but failed to enact substantive changes. One participant (PSW, Female) highlighted this disparity, stating, *“We are overworked, and let us say payment is less…I think workload management could potentially be an area that needs to be improved.”* Participants also observed that the principles outlined in the *National Standard of Canada for Psychological Health and Safety in the Workplace* (30) were seldom prioritized in LTC settings. To address these challenges, participants proposed small, actionable steps to enhance mental well-being and foster work-life balance, aiming to create meaningful change. For example, one participant (Manager, Male) advocated for policies encouraging employees to disconnect from work, stating: *“There should be policies that will encourage employees to disconnect from work.”* Without such policies, HCWs found it challenging to separate themselves from job responsibilities, leading to heightened stress, burnout, and a decline in overall well-being.

### 3.4 The need for a context-specific tool to assess burnout in LTC settings

A consistent theme among participants was the urgent need for a context-specific tool to assess burnout in LTC settings. In critically reviewing existing questionnaire items, participants prioritized excluding those considered overly triggering or emotionally distressing. Questions addressing suicide or self-harm were identified as particularly concerning, given their potential to exacerbate fragile mental health. In scenarios such as research, where interventions or resources may be limited, such items could worsen psychological distress, particularly if individuals perceive inadequate support. Participants proposed adopting *“gentle language when discussing death and coping strategies”* to mitigate potential harm and foster a supportive approach. Reflecting on the sensitivity of addressing mental health, one participant (Manager, Female) noted, *“I still think it’s uncomfortable to ask these questions, but it might not be a bad idea to know where people are at.”* This quote reflects the delicate balance between addressing mental health sensitively and the necessity of understanding and supporting HCWs in challenging workplace environments.

To enhance the tool’s relevance to LTC settings, participants suggested reorganizing the questionnaire to align with distinct domains of health and well-being—such as emotional, physical, occupational, social, spiritual, intellectual, environmental, and financial dimensions. Questions eliciting strong emotional responses could be grouped under specific categories, thereby improving the tool’s structure and contextual sensitivity. These modifications were seen as crucial for tailoring the tool to the distinct needs of LTC environments. While the restructuring of the questionnaire sparked significant discussion, participants expressed little concern about its composite design, which incorporated items from multiple instruments. Although time constraints were acknowledged, concerns about the questionnaire’s length were minimal.

## 4 Discussion

This manuscript presents preliminary findings from the initial two phases of the HH project, utilizing a co-design approach to gain deeper insights into the experiences of HCWs, including leadership, within LTC settings. This study is the first of its kind to adopt a co-design approach to examine the pervasive issue of mental ill-health and burnout in LTC, leveraging the perspectives of frontline workers to inform the development of a Burnout Assessment Tool (BAT) tailored to this unique context. Our findings underscore the persistent challenges faced by HCWs and leaders, which contribute significantly to burnout and mental distress. In alignment with Camero and Carrico (32), participants highlighted various workplace pressures—such as excessive workload, inadequate managerial support, and limited healthcare human resources—as primary contributors to stress, anxiety, and burnout. Many emphasized the detrimental impact of these high demands on their health and well-being. Notably, both frontline and non-frontline participants reported experiencing significant levels of burnout, a finding consistent with a systematic review and meta-analysis on burnout among healthcare workers during COVID-19 (33). Furthermore, the perceived failure of employers to uphold the *National Standard of Canada for Psychological Health and Safety in the Workplace* (30) was identified as an exacerbating factor.

To address these challenges, participants advocated for systemic reforms, including work-life balance policies, improved workload management, and pay equity, alongside low-cost interventions such as fostering gratitude, appreciation, and recognition. These measures not only strengthen interpersonal relationships but also enhance well-being and morale. A key priority highlighted was the development of a context-specific burnout assessment tool to enable early detection and timely intervention. Such strategies align with the Quadruple Aim and *The Future of Nursing 2020–2030* recommendations (34). Globally, addressing these concerns is particularly pressing in the face of an aging population, contributing directly to the Sustainable Development Goals—SDG 3 (Good Health and Well-being) and SDG 8 (Decent Work and Economic Growth) (35).

### 4.1 The co-design process framework

The co-design framework employed in this study was central to addressing these priorities, fostering collective learning and ensuring the meaningful engagement of HCWs throughout the research process. By prioritizing participant expertise, this collaborative approach aligns research objectives with lived experiences, ensuring the development of practical and relevant solutions. Engaging stakeholders from the project’s inception (Phases 1 and 2) through to its intended conclusion further guarantees that the resulting tool is both effective and applicable. This innovative approach holds promise for reshaping how burnout and mental health challenges are identified and managed in LTC settings.

As the current analysis focuses on the first two phases of the co-design process framework (pre-design and generative), it provides a unique opportunity to consider the value of using this process within LTC. By involving participants in decision-making, co-design empowered them to identify and inform potential strategies that can improve their workplace culture and beyond (27). In this study, the co-design process typifies a robust methodology for engaging stakeholders, particularly HCWs, and is well-supported in the literature, spanning multiple disciplines and proving its value in engaging stakeholders (21, 36). The implementation of co-design in this research is particularly relevant as it facilitated the incorporation of diverse viewpoints, which ensures that the resulting BAT will accurately capture the experiences of HCWs, context-appropriate, and culturally sensitive (26).

Another significant contribution of the HH project is its use of virtual methods, aligning with the broader trend toward digital co-design. The virtual platform ensured accessibility, enabling participation from diverse geographical locations, time zones, and those with physical limitations, thereby fostering a more inclusive range of perspectives. It also reduced costs associated with travel and logistics while providing scheduling flexibility for busy participants, such as HCWs. This created a comfortable environment for more open and diverse input. Additionally, the platform facilitated real-time collaboration, idea sharing, and iterative design, while automatically recording discussions, streamlining data collection and analysis. In this study, virtual co-design proved especially valuable during COVID-19 restrictions, maintaining momentum in research despite logistical challenges. The use of co-design in a virtual environment underscores the evolving landscape of digital collaboration demonstrating its adaptability to contemporary challenges, such as the COVID-19 pandemic. The literature on digital co-design highlights the growing use of online platforms, social media, and collaborative digital tools to engage stakeholders remotely (37). The effectiveness of virtual focus groups and online collaboration in this study adds to the expanding body of evidence supporting digital co-design as a viable and beneficial approach. Sanz et al. (38) identified key aspects of the digital co-design process that contribute to seamless engagement and successful outcomes, further validating its potential. As a result, digital co-design emerges as a valuable tool in healthcare research, offering enhanced accessibility and flexibility.

#### 4.1.1 Development of the BAT

In this study, the co-design process cultivated a sense of commitment to the outcomes, increasing the likelihood of successful implementation of the BAT and its sustainability. For example, participants expressed a sense of trust, as their feelings were validated through shared experiences. They found the process therapeutic, enabling them to voice their concerns and contribute to essential changes within the sector. Through this collaborative process, participants identified key areas for intervention, including staffing, pay equity, opportunities for both worker and leadership training and education, concrete measures to address discrimination and racism, and strategies to mitigate workplace violence. Upon reviewing the questionnaires, participants opted to exclude items that were particularly triggering or evoked strong emotional responses. This decision suggests that more conservatively framed assessment tools, like the WHO-5 Well-being Index or Warwick-Edinburgh Mental Wellbeing Scale, may be preferable—especially in settings with limited access to training and support resources.

#### 4.1.2 Challenges of the co-design approach

The literature identifies certain challenges in co-design, such as accessing resource-constrained individuals, inconsistent participation, power imbalances, and misalignment of goals (39). Resource limitations and lack of organizational support further hinder optimal solution implementation. As highlighted in this study, maintaining a diverse pool of participants with varying roles and characteristics was a common challenge. Ensuring equitable participation and representation of all stakeholders—particularly certain groups of HCWs (i.e., PSWs)—and incorporating diverse perspectives is essential but logistically challenging due to the varying groups and career stages of participants (40). Managing power dynamics in the co-design process is also critical to avoid reinforcing existing hierarchies (41). We agree with Jagtap (39) who found that individuals perceived as knowledgeable or authoritative often dominate discussions, aligning outcomes with their preferences. In our study, we addressed this by conducting tailored sub-group sessions with specific HCWs to better understand their distinct needs. This approach allowed participants to openly voice concerns within smaller, safer groups, fostering a more equitable environment. Participants expressed appreciation for the opportunity to share both in larger discussions and in intimate settings where they felt more comfortable. Additionally, while differing opinions can complicate decision-making in co-design, this issue was not prominent in our study. Despite the challenges, the advantages of co-design in improving the quality and relevance of research outcomes make it a desirable methodological approach for future studies.

### 4.2 Strengths and limitations

This study is the first to use a co-design approach to examine burnout among HCWs and leaders in Ontario’s LTC sector. In this study, the virtual co-design offered significant advantages. It was cost-effective, accessible, and inclusive of diverse perspectives, allowing for collaboration across different HCWs/stakeholders, including community members, end-users, and marginalized groups. This approach ensures that the final design (i.e., the BAT) reflects the needs, experiences, and insights of all involved, leading to more equitable and sustainable outcomes. The collaborative nature of the co-design process fosters creativity and innovation, generating new ideas that may not arise in traditional design processes (42, 43).

Despite its strengths, the study has limitations to consider when interpreting the findings. A key challenge was participants’ time constraints during the co-design process. Data collection occurred at the height of the pandemic, a period when HCWs and leaders were overwhelmed with responsibilities. This considerable burden made it challenging to allocate time for the two-hour meetings, ultimately contributing to the relatively limited sample size. Although the virtual co-design process offered several advantages, it posed challenges, such as participant fatigue from prolonged screen time, technological interruptions, and overlapping voices during discussions.

To mitigate these, we limited focus group sessions to 2 h with scheduled breaks and provided asynchronous options, such as email feedback or collaboration tools, for flexible participation. A co-host moderator swiftly managed technological issues, and staggered participation allowed for engagement at different stages of the design process, accommodating diverse schedules. We focused on clinical staff to ensure participants had the requisite knowledge of the 13 psychosocial risk factors (30) to guide evidence-based strategies and interventions for enhancing psychological health and safety among HCWs. However, including non-clinical staff, such as housekeeping, could have brought historically overlooked perspectives to the discussion.

### 4.3 Implications for practice, policy and future research

The implications of this study underscore the critical need for targeted strategies to address the root causes of burnout and improve the work environment for HCWs, particularly in LTC settings (3, 4, 44). A context-sensitive assessment tool, such as the Burnout Assessment Tool (BAT), emerges as a pivotal instrument for identifying risks and understanding the unique challenges faced by HCWs and leaders. Its implementation not only facilitates early detection of burnout but also informs interventions that address systemic gaps, ultimately fostering healthier work environments. Integrating such tools into existing wellness initiatives, like stress management programs, offers a pathway for systemic reforms rooted in real-world experiences. This alignment supports LTC organizations in fulfilling their ethical responsibility to safeguard employees’ well-being while enhancing care quality for residents (4). Moreover, regular assessments guided by the BAT promote open communication, inform policy decisions, and ensure regulatory compliance, contributing to cost-effective, sustainable improvements in LTC settings.

The engagement of both leadership and frontline staff during implementation is essential for ensuring the relevance, practicality, and sustainability of these interventions. Co-designed strategies based on the lived experiences of HCWs not only strengthen mental health and retention but also enhance the overall quality of care provided to LTC residents. Beyond immediate organizational benefits, these findings have broader implications for shaping policies and practices across the global north, where similar challenges exist. Furthermore, the study lays a foundation for future research aimed at evaluating the effectiveness of these interventions in reducing burnout. Insights gained can drive innovations in addressing HCW distress across diverse high-stress healthcare environments, contributing to the development of adaptable and scalable solutions to a global challenge.

### 4.4 Recommendations for future research

Based on our findings, we recommend that future co-design projects incorporate preparatory sessions to familiarize participants with the methodology, especially those new to co-design. Although time constraints in this study precluded a pre-meeting, such sessions are essential for addressing methodological challenges, including ethics, and ensuring participants’ perspectives are accurately captured (45). Researchers should develop “engagement literacy”—skills in communication, facilitation, and conflict resolution—while recognizing the additional time demands of co-design. Research managers must also acknowledge the value of relational knowledge fostered by co-design and provide support by facilitating conflict resolution and accommodating longer project timelines.

Preparatory meetings are also crucial for clarifying participant expectations and fostering meaningful engagement (46). Clear instructions and reference materials should be provided to maintain consistency and align the co-design process with study objectives. For instance, in this study, participants were given sample questionnaires to review before focus group sessions, which enhanced their contributions and ensured cohesive, relevant insights (47). Flexibility in scheduling meetings is another critical factor, allowing participants to balance the demands of their roles in LTC settings. This approach acknowledges the dynamic nature of HCWs’ responsibilities and facilitates a more in-depth exploration of their experiences (12). Additionally, leveraging advanced and freely available qualitative tools, such as Delve, can improve the management and analysis of the substantial data generated through co-design. These tools enable systematic and rigorous identification of themes and patterns, enhancing the efficiency and depth of analysis. Together, these recommendations serve as a strategic roadmap for optimizing the co-design process in research on complex, high-pressure environments like LTC.

Efforts should focus on ensuring diverse participant perspectives, particularly from equity-seeking groups. Considerations such as profession or occupation, years of experience, geographical location, and familiarity with workplace psychosocial risk factors (e.g., engagement and workload management) are critical. Adequate representation enriches discussions, incorporating varied experiences and fostering strategies to address challenges, such as participant availability, encountered in this study.

Future co-design studies should prioritize intersectional factors, including gender, ethnicity, and race, in participant recruitment. Inclusive approaches—such as intersectional strategies, flexible participation formats (e.g., hybrid and staggered sessions), and creating safe, inclusive spaces for marginalized voices—are essential for ensuring diverse representation. Offering financial compensation or technical support further enhances accessibility and equity. These measures help uncover the nuanced impacts of these factors on HCWs’ experiences in the evolving LTC landscape. Engaging stakeholders or healthcare representatives prior to the co-design process is vital for aligning with its participatory nature (48). Early stakeholder input addresses community interests and enhances study relevance (46). Additionally, Sanders and Stappers’ (27) co-design framework offers a valuable and adaptable methodology that enhances coherence and depth across diverse healthcare contexts. Further applied research is needed to explore HCWs’ contributions to co-design, including the integration of digital and non-digital approaches. Adopting these strategies in future research can refine and enhance the co-design process, ensuring its effectiveness across diverse healthcare settings and maximizing its impact.

## 5 Conclusion

This study, conducted as part of a larger Healing the Healers project, explored the experiences of HCWs and leaders in the LTC sector, where burnout and distress are a pressing concern. Insights from the study’s initial phases identified key thematic drivers of distress for HCWs, which will inform the development of a burnout assessment tool, laying the groundwork for targeted interventions. Using a co-design approach, this research contributes valuable insights to healthcare literature, bringing attention to perspectives often overlooked in policy decisions. Despite challenges, co-design proved to be an effective, collaborative method for developing inclusive solutions to address burnout in LTC settings. These insights underscore the potential of co-design in future research within this context, offering lessons on both the strengths and limitations of this approach.

## Data Availability

The raw data supporting the conclusions of this article will be made available by the authors, without undue reservation.
